# Exploring Knowledge, Attitudes, and Practices Regarding Dengue Fever Among University Students in Bangladesh: A Cross‐Sectional Study

**DOI:** 10.1002/hsr2.71714

**Published:** 2025-12-30

**Authors:** Nasimul Ghani Usmani, Pradip Chandra, Tamanna Hassan, Sourav Chandra Debnath, Sunjida Munmun Md, Bony Amin, Sirajul Islam Pial Md, Hasanuzzaman Md, Asaduzzaman Miah Md, Md. Hafizur Rahman, Nazmul Hassan

**Affiliations:** ^1^ Department of Environmental Sanitation Patuakhali Science and Technology University (PSTU) Patuakhali Bangladesh; ^2^ Maternal and Child Health Division, International Centre for Diarrhoeal Disease Research, Bangladesh (icddr,b) Dhaka Bangladesh; ^3^ Department of Applied Food Science and Nutrition Chattogram Veterinary and Animal Sciences University (CVASU) Chattogram Bangladesh; ^4^ Department of Human Nutrition and Dietetics Patuakhali Science and Technology University (PSTU) Patuakhali Bangladesh; ^5^ Department of Biochemistry and Food Analysis Patuakhali Science and Technology University (PSTU) Patuakhali Bangladesh; ^6^ Department of Entomology Patuakhali Science and Technology University (PSTU) Patuakhali Bangladesh

**Keywords:** attitudes and practices, Bangladesh, dengue fever, KAP, knowledge, university students

## Abstract

**Background and Aims:**

Inadequate knowledge, attitudes and practices (KAPs) related to dengue fever (DF) can substantially affect vulnerable people, such as Bangladeshi university students, because of their living status. However, this topic has never been explored. Therefore, this study was conducted to assess the levels and factors influencing knowledge, attitudes and practices of university students in Bangladesh.

**Methods:**

A cross‐sectional study was conducted from September 2023 to January 2024 among 671 university students through convenient sampling. Logistic regression models were employed to analyze the association between outcome measures across sociodemographic and academic variables.

**Results:**

Around one‐third (30.40%) of the university students had good knowledge of DF, while just over one‐third (35.90%) had good practices and nearly three‐fourths (72.30%) demonstrated a higher attitude towards DF. Participants who had a dengue‐related subject in their curriculum were more likely to have higher knowledge (AOR: 1.7, 95% CI: 1.18–2.45) and a positive attitude (AOR: 1.55, 95% CI: 1.07–2.28) toward dengue fever. Participants who encountered dengue cases within their family members (AOR: 1.68, 95% CI: 1.18–2.4) showed significant preventive practices. However, the likelihood of adopting preventive practices was lower when dengue cases were reported among neighbors (AOR: 0.57, 95% CI: 0.37–0.88). Moreover, robust preventive practices were significantly associated with good knowledge levels (OR: 1.75, 95% CI: 1.25–2.46), while a partially significant relationship was observed between preventive behaviors and a positive attitude among participants (OR: 1.47, 95% CI: 1.00–2.16).

**Conclusions:**

Overall knowledge and preventive practices concerning dengue fever among university students were found to be inadequate. Raising awareness should involve coordinated efforts by university authorities and family members. Furthermore, translating knowledge and attitude into preventive actions is crucial to reduce risks of dengue fever among these vulnerable groups.

**Abbreviations**
AOR: adjusted odds ratio
BMRC: Bangladesh Medical Research Council
CDC: Centres for Disease Control and Prevention
CFR: crude fatality rate
COR: crude odds ratio
DENV: dengue virus
DF: dengue fever
HL: Hosmer–Lemeshow test
KAP: knowledge, attitudes, and practices
VIF: variance influence factor
WHO: World Health Organization

## 1 Introduction

Dengue is a rapidly escalating global health concern, with nearly half the world's population at risk and endemic transmission in 108 countries. In 2019, the World Health Organization (WHO) reported over five million dengue infections, reflecting a tenfold increase compared to 2000 [1]. The year 2023, witnessed an unprecedented surge in dengue incidence, affecting more than 80 countries across all WHO regions. Persistent transmission, coupled with an unexpected spike in cases since early 2023, has resulted in over 6.5 million reported infections and more than 7300 dengue‐related deaths [2]. Dengue virus remains (DENV) endemic primarily in Latin American, Caribbean countries, and South Asia, with Asia bearing 70% of the global disease burden.

Dengue outbreaks in Bangladesh have intensified in recent years, driven by a confluence of factors including rapid urbanization, climate change, and extended monsoon seasons. Household hazards have further facilitated the proliferation of Aedes mosquitoes, contributing to recurrent large‐scale outbreaks [3, 4]. A significant endemic occurred in 2019, followed by a marked resurgence in 2023, affecting several countries across Asian [5]. In Bangladesh, dengue cases rose sharply from 62,382 in 2022 to 308,167 in 2023, with associated deaths increasing from 281 (CFR 0.45%) to 1598 (CFR 0.52%) over the same period [6].

The rising incidence of mosquito‐borne diseases is predominantly anthropogenic, driven by climate change factors like high temperatures and excessive rainfall, which impact mosquito bionomics [7]. Nonetheless, targeted human interventions and behavioral modifications can substantially reduce dengue transmission [8]. Public education and awareness campaign on vector control improve attitudes and promote protective practices [9].

Knowledge, attitudes, and practices (KAPs) are foundational constructs in public health, underpinning efforts in disease prevention and health promotion. The KAP model offers a structured framework for understanding health behaviors, enabling effective intervention design and evaluation [10, 11]. The US Centres for Disease Control and Prevention (CDC) underscores the utility of KAP surveys during public health emergencies, where tailored responses are essential to address the diverse needs of affected populations [12]. Moreover, KAP studies serve as critical tools in assessing the effectiveness of public health initiatives and enhancing strategies to optimize health outcomes [13].

The university setting plays a pivotal role in shaping students' personal, social, and intellectual development, influencing their values, decision‐making, and long‐term societal contributions [14]. University students, irrespective of their academic discipline, represent a strategically important demographic whose decisions can affect future disease transmission patterns [15]. As accessible and informed members, students can be mobilized through academic institutions and digital platforms to disseminate knowledge within their communities [16]. With sufficient awareness and positive health attitudes, they can promote preventative practices, contributing to public health initiatives, including dengue control efforts.

Several studies have been conducted in Bangladesh to examine the influence of KAP on dengue fever and mosquito control, including one that specifically examined the KAP of students residing in Dhaka city [17, 18, 19]. However, these studies lack key variables such as dengue infection history among students, household members, and neighbors' factors that may shape student's KAP profile. A study reported that due to their movement across the country for educational or familial reasons, may act as an inadvertent carriers of the dengue fever (DF) virus [20]. This potential for dengue transmission to non‐endemic regions in Bangladesh has become evident, particularly following three consecutive outbreaks in 2019, 2021, and 2022. During the 2019 outbreak, the most extensive to date nearly half (48.4%) of all reported cases were distributed across all 64 districts of Bangladesh. Similarly, in 2021, 20.4% of cases were recorded from areas outside Dhaka [20]. The actual scale of the 2021 outbreak, which involved 28,429 cases and 105 deaths, remains uncertain due to the concurrent COVID‐19 pandemic [21]. In 2022, over one‐third (37.5%) of reported cases originated from regions beyond Dhaka city.

Evidence indicates that dengue transmission is occurring locally in Cox's Bazar which accounted for 42.7% (1466 out of 3427) of all reported cases in the Chittagong division [20]. Recent outbreak data suggest that non‐endemic southern districts such as Jessore, Pabna, and Cox's Bazar are emerging as potential dengue hotspots. One study reported a nearly 24‐fold decline in dengue incidence in Dhaka 2 weeks after the Eid exodus during the 2019 outbreak, accompanied by a four‐ to seven‐fold increase in cases in certain southern districts [22]. Notably, this elevated incidence in regions outside Dhaka persisted until the end of the outbreak, indicating local dengue transmission [22]. Supporting this, a multi‐centered hospital‐based study identified 262 district‐level dengue cases in Dhaka hospitals with no recent travel history and local dengue transmission was also confirmed in a northern district during the same outbreak [23, 24]. Collectively, these findings underscore the geographic expansion of dengue into non‐endemic areas in Bangladesh.

In 2023, the highest burden of dengue was observed among individuals aged 21–25 years (30,415 cases) and 26–30 years (26,492 cases) [5] age groups that encompass the majority of university students [25, 26]. Given their mobility for educational and familial reasons, students residing in dormitories or rented accommodations may serve as key vectors in the dissemination of dengue virus across regions. Despite this, no comprehensive study has been yet assessed the KAP profile of university students in Bangladesh in the aftermath of these outbreaks.

Given their high mobility and social connections, which increase their risk of exposure to dengue virus [20], it is essential to conduct a KAP study among university students in Bangladesh. This study aims to assess their knowledge, attitudes, and practices to dengue, thereby identifying gaps and misconceptions that could inform the development of targeted educational campaigns and preventive strategies. The findings would provide valuable insights for policymakers and public health authorities, supporting the formulation of effective dengue control measures and contributing to the reduction of future outbreaks. Moreover, engaging students in this study fosters community level awareness and participation in public health initiatives, reinforcing a comprehensive approach to dengue prevention.

## 2 Materials and Methods

### 2.1 Study Design, Area, and Participants

A cross‐sectional study of these university students was conducted between September 2023 and January 2024. This study utilized a self‐reported online survey to evaluate dengue responses through a KAP study among university students in Bangladesh (Data collection universities are included in Supporting Information S1: Appendix 3.

### 2.2 Sampling Method

According to Bangladesh Education Statistics, in 2022, there are approximately 164 (public and private) educational institutions where the total number of students was 1,034,320 [27]. Participants were recruited through convenience sampling technique.

### 2.3 Sample Size

The sample size was calculated via the following Cochran's formula: *n *= *z* ^2^ *pq*/*d* ^2^,

$$n=\frac{z^{2}\text{pq}}{d^{2}};n=\frac{1.96^{2}\times 0.68\times [1-0.68]}{0.05^{2}}=334.37\approx 335$$

where *n* = total number of samples, *z *= the statistic that corresponds to the level of confidence = 1.96 [95% confidence level], *p* = a measure of expected prevalence, 68.32%, as there is a previous study in Bangladesh conducted among university students residing in Dhaka city, *q* = (1−*p*), *d *= precession of the prevalence estimate (10% of 0.5); then, the sample size was calculated as 335 subjects [20]. A 10% nonresponse rate was considered and the optimal sample size of 370 was determined. However, to improve the external validity and generalizability of the research, 671 samples were taken, which was greater than our estimated number [28].

### 2.4 Data Collection Tools and Measurements

The structured questionnaire (in both the native Bengali and English languages) was developed in Google forms after a literature review, which was adapted from previous studies [18, 20, 29]. The questionnaire was carefully evaluated for content validity before data collection. Content Validity Ratio (CVR) was assessed with the guidance of two expert supervisors. Each item was reviewed for essentiality, clarity, and relevance. Based on their recommendations, minor adjustments were made, and all items were subsequently accepted as appropriate. The questionnaire was translated from English to Bangla using a forward translation approach, followed by expert review with two supervisors. All items were carefully reviewed and adjusted to ensure clarity, cultural relevance, and conceptual equivalence (Full questionnaire has been provided in the Supporting Information S2: Appendix 1).

The internal consistency reliability of the questionnaire, evaluated using Cronbach's alpha test, resulted in a value of 0.80, indicating good reliability. A 80% cut‐off scores was used for categorizing KAP levels as “good” or “poor” since it has been consistently used in similar studies both within the country as well as in related research conducted in other countries assuring established standards, facilitating comparability across different research contexts [20, 29, 30, 31]. It has three sections with response items: sociodemographic and academic section; dengue infection history of family members, neighbors and self‐dengue infection history; and the final KAP section. In sociodemographic and academic section, participants living in different residential units. Residential units are categorized as high‐rise buildings (greater than 5 stories); low rise building (lower or equal to 5 rise building); mixed: shop, factory, office, and residence in the same building. Living with their family members, major department (Arts and Social Sciences, Business Studies, Science and Engineering) are included in the study [20].

Knowledge was evaluated through 24 dichotomous (yes/no) questions. Each correct response was assigned 1 point, while incorrect responses received 0 points. Scores between 0 and 18 were categorized as “poor” knowledge and scores from 19 to 24 classified as “good” knowledge. Attitude was assessed using six items rated on a five‐point likert scale (1 = strongly disagree, 2 = disagree, 3 = neutral, 4 = agree, 5 = strongly agree). The total attitude score ranged from 0 to 30. Scores between 0 and 23 were considered indicative of poor attitude, while scores from 24 to 30 represented as good attitude. Practice was measured using 21 dichotomous (yes/no) questions on preventive practices. Correct answers received 1 point, while incorrect answers received 0 point. Scores between 0 and 16 indicating “poor” practice and scores from 17 to 21 reflect “good” practice. (The KAP questions and their corresponding responses are provided in Supporting Information S1: Appendix 1).

### 2.5 Data Collection Strategy

A structured questionnaire (in both the Bangla and English languages) was used for data collection. During the data collection period, the authors personally visited classes at several nearby universities and provided the survey link to students. Before data collection procedures, the authors explained the purpose of the study and described the contents of the questionnaire to facilitate understanding. For universities that the authors could not visit in person, the respective class representatives were contacted. These representatives shared the Google Form link with their classmates, explained the questionnaire content, and collected responses at a suitable time. All responses were submitted through the survey link and automatically recorded in Google Forms. The authors subsequently reviewed collected data for completeness and accuracy before analysis, ensuring the reliability and integrity of the dataset.

A preliminary assessment was conducted with 25 participants from the designated population to evaluate the questionnaire's acceptability and clarity. However, during the pilot testing, some participants struggled with the questionnaire in the English version. To improve comprehension, the questionnaire was translated into Bengali, and both versions were included in the final survey. This change addressed misunderstandings that led to inaccurate responses. The pilot results showed that the bilingual format reduced language barriers, leading to more accurate responses. To avoid bias, pilot study participants were excluded from the final data collection. The initial page of the questionnaire included an informed consent statement that outlined the study's objectives, procedures, and participants' right to decline participation.

### 2.6 Statistical Analysis

The obtained data were rechecked for completeness and consistency. Microsoft Excel and STATA 17 software (College Park, Texas) were used for data management and statistical analysis, respectively. Descriptive statistics (frequency and percentage) were performed, and the Pearson's *χ* ^2^ test of independence was tested to determine the associations in between the socio demographic variables and the dependent variables—knowledge, attitudes, and practice variables (The association is provided in the Supporting Information S1: Appendix 2). Normality of the data was assessed using the Shapiro–Wilk test, indicating a normal distribution (*p* > 0.05). To check the multicollinearity of the variables, the variance influence factor (VIF) of the variables less than two was used for multiple logistic regression analysis [32] (VIF values are provided in the Supporting Information S1: Appendix 5). Effect sizes were reported as *R*² values (*R*² values are provided in the Supporting Information S1: Appendix 4). Unadjusted (Crude odds ratio, COR) and adjusted logistic regression (Adjusted odds ratio, AOR) analyzes were performed to determine the predictors of the KAP level. Multiple logistic regression analysis of knowledge, attitude, and practices with predictors of dengue fever were performed at the 0.05 α level. The goodness of fit results for knowledge (*p* value = 0.5837), attitudes (*p* value = 0.8026) and practices (*p* value = 0.1328) was assessed through the Hosmer–Lemeshow (HL) test.

### 2.7 Ethical Consideration

Written informed consent was obtained from all participants prior to data collection, in accordance with the ethical guidelines of the WHO and the Bangladesh Medical Research Council (BMRC). Research Ethics Committee (REC) of the Department of Environmental Sanitation, Patuakhali Science and Technology University, Bangladesh reviewed and approved the study protocol (Approval No.: PSTU/IEC/2023/72).

## 3 Results

### 3.1 Sociodemographic and Academic Information of the Participants

Majority of the participants in this study were male (67.7%) and lived with their families (59.5%). Nearly three‐fourths (73.47%) of the participants were from the Science and Engineering department. A significant proportion of participants reported having dengue‐related subjects in their curriculum (43.7%). Around one in ten (10.6%) participants had experienced dengue infection in the past 6 months, while over one‐third (37.7%) reported dengue cases among their household members and more than two‐thirds (69.75%) reported cases in their neighborhoods (Table 1).

**Table 1:** Sociodemographic and academic information of the participants (*N* = 671).

Variables		Frequency (*n*)	Percentage (%)
Gender	Male	454	67.7
	Female	217	32.3
Living with family	Yes	399	59.5
	No	272	40.5
University type	Public	571	85.10
	Private	100	14.90
Department/faculty/major	Arts and social sciences	148	22.1
	Business studies	30	4.5
	Science and engineering	493	73.5
Dengue‐relevant subject in the curriculum	Yes	293	43.7
	No	378	56.3
Heard about dengue	Yes	637	94.9
	No	34	5.1
Dengue infection history in last 6 months	Yes	71	89.4
	No	600	10.6
House members infected with dengue in last 6 months	Yes	253	37.7
	No	418	62.3
Neighbors infected with dengue in last 6 months	Yes	468	69.8
	No	203	30.3

### 3.2 Overall Scores of Knowledge, Attitudes, and Practices Towards Dengue Fever of University Students

**Figure 1:**
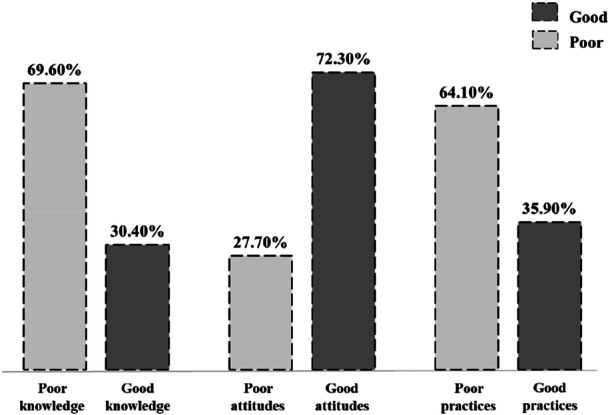
Overall scores of knowledge attitudes and practices towards dengue fever.

Figure 1 illustrates the overall scores of knowledge attitudes and practices towards dengue fever among university students. About one‐third (30.40%) of university students had good knowledge of DF, and just over one‐third (35.90%) exhibited good practice levels. Nearly three‐fourths (72.30%) showed good attitudes towards dengue.

### 3.3 Multiple Logistic Regression Analysis of Knowledge, Attitude, and Practices With Predictors of Dengue Fever

The multiple logistic regression analysis in Table 2 revealed that participants with dengue‐related subject in their current curriculum were nearly two times more likely to have adequate knowledge (OR: 1.70, 95% CI: 1.18–2.45, *p* = 0.004) and a positive attitude (OR: 1.55, 95% CI: 1.07–2.28, *p* = 0.025) on dengue fever. Participants whose family members had experienced dengue fever were 68% more likely to engage in preventive practices (OR: 1.68, 95% CI: 1.18–2.40, *p* = 0.004). However, those reporting dengue cases in their neighborhood were nearly two times less likely to adopt preventive measures (OR: 0.57, 95% CI: 0.37–0.88, *p* = 0.011).

**Table 2:** Multiple logistic regression analysis of knowledge, attitude, and practices with predictors of dengue fever.

Variables	Knowledge		Attitude		Practice	
	aOR (95% CI)	*p* value	aOR (95% CI)	*p* value	aOR (95% CI)	*p* value
Gender						
Male	1		1		1	
Female	1.13 (0.78–1.63)	0.529	0.63 (0.43–0.91)	0.015	1.32 (0.92–1.89)	0.131
Living with family						
No	1		1		1	
Yes	1.04 (0.73–1.50)	0.817	0.95 (0.66–1.38)	0.787	1.16 (0.82–1.64)	0.401
Residential unit						
Mixed	1		1		1	
High‐rise	1.13 (0.66–1.92)	0.662	1.53 (0.90–2.62)	0.114	1.29 (0.78–2.12)	0.322
Low‐rise	1.28 (0.79–2.07)	0.324	1.24 (0.79–1.96)	0.355	1.15 (0.73–1.79)	0.548
Department/faculty/major						
Business studies	1		1		1	
Arts and social sciences	0.85 (0.32–2.28)	0.75	0.96 (0.38–2.41)	0.929	2.71 (1.03–7.15)	0.044
Science and engineering	1.06 (0.44–2.58)	0.896	1.47 (0.65–3.33)	0.36	2.40 (0.99–5.77)	0.05
Dengue‐relevant subject in the curriculum						
No	1		1		1	
Yes	1.70 (1.18–2.45)	0.004	1.55 (1.07–2.28)	0.025	1.20 (0.84–1.71)	0.312
Dengue infection history in last 6 months						
No	1		1		1	
Yes	0.83 (0.46–1.50)	0.532	1.03 (0.55–1.93)	0.934	0.63 (0.35–1.11)	0.111
House members infected with dengue in last 6 months						
No	1		1		1	
Yes	0.88 (0.60–1.28)	0.493	1.26 (0.86–1.86)	0.237	1.68 (1.18–2.40)	0.004
Neighbors infected with dengue in last 6 months						
No	1		1		1	
Yes	1.40 (0.91–2.13)	0.125	1.43 (0.91–2.25)	0.125	0.57 (0.37–0.88)	0.011

### 3.4 The Associations Between Dengue Knowledge and Practices, Knowledge and Attitudes, Attitudes and Practices of University Students

**Figure 2:**
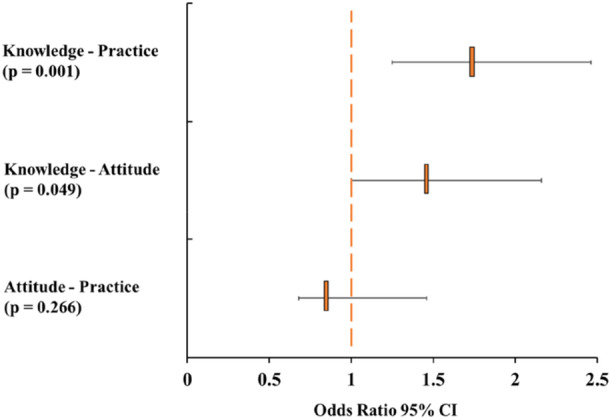
Associations among dengue knowledge, attitudes, and practices.

Figure 2 illustrates the associations between dengue related knowledge, attitudes, and preventive practices among the study participants. Preventive practices were found significantly associated with good knowledge levels among university students (OR: 1.75; 95% CI: 1.25–2.46). In addition, partial significant association was observed among participants with good knowledge (OR: 1.47; 95% CI: 1.00–2.16) and positive attitudes. However, no statistically significant association was observed between positive attitudes and preventive practices among students (OR: 0.86; 95% CI: 0.68–1.46).

## 4 Discussion

To our knowledge this is the first study which explores the knowledge, attitudes and prevention practices towards DF among university students in Bangladesh. According to the study, around one third (30.4%) of participants demonstrated high knowledge scores. This proportion is lower than those reported in comparable studies conducted in India Pakistan, Malaysia, Brazil and Jamaica [33, 34, 35, 36, 37]. Such variation may reflect differences in DF awareness, varying research sites, methodologies, and sample sizes. Notably, a 2021 study conducted among university students in Dhaka city reported higher knowledge scores (66.72%) [20]. The observed decline in knowledge levels over a 3‐year period is concerning, particularly given the heightened vulnerability of this demographic.

Blood transfusion has been recognized as a possible mode of dengue fever transmission among these learner group [38, 39, 40, 41]. About half of the participants in our study (48.1%) stated that dengue fever could be transmitted through blood transfusion from a dengue infected person to a healthy human being. In line to our findings, study conducted in Jamaica had 45% response to dengue transmission through blood transfusion [37]. However, higher response rate had also been found in others studies conducted in Yemen, Nepal [29, 42]. Moreover, university students of our country have good practices towards blood donation to mass population [43, 44]. Our study insists for frequent check‐up for dengue test among university students and take preventive measures through campaign, social media, and raising awareness in collaboration with local people.

Poor knowledge has also been found among university students regarding correct time of feeding habit of the Aedes mosquito. Our study revealed, only 35.2% of the students were aware about the biting habit of the dengue vectors during morning/evening where the percentage declined from the previous findings (74.1%) conducted in Bangladesh in the year 2021 [20]. Similar findings were also reported in studies conducted in Jamaica [37] and Malaysia [45]. In contrast to our findings, increased awareness on dengue biting time was found higher in studies, conducted in Thailand, Srilanka, Yemen, and Nepal [29, 42, 46, 47]. This indicates the importance of introducing practical knowledge‐based programs among university students to prevent and control disease spread.

High attitude score (72.28%) observed among students suggests a heightened level of concern regarding DF. The profound impact of the COVID‐19 pandemic may have contributed to heightened awareness and more favorable attitudes toward infectious diseases in general [20]. As a result, majority of students in our study perceived dengue fever as a serious health concern and expressed support for its prevention and control efforts. However, such attitudes must be effectively translated into tangible preventive actions. This finding is consistent with previous studies conducted in Yemen, Nepal, Malaysia, and among university students in Dhaka city [20, 29, 45, 48]. Moreover, such elevated concern may be attributed to increased exposure to information on DF and its associated risks, disseminated through educational institutions, social media platforms, and broadcasting of news and information through television channels and print journalism.

In our study, 48% of the participants did not consider them at risk to dengue infection. Coherent attitude has also been noticed in Nepal and Venezuela [29, 49]. This perception may stem from limited personal exposure to dengue cases within their families, immediate surroundings, communities or in academic residence, where reports of severe dengue infections have been relatively rare.

Approximately one‐third (35.92%) of the participants in our study reported high practice scores, a finding consistent with studies conducted in Jamaica, Nepal, and Indonesia [29, 31, 37]. However, this proportion is lower than that reported among university students in Malaysia [45]. A possible explanation might be that the students may not perceive themselves to be at risk of contracting DF, which could diminish their motivation to adopt preventive behaviors. In addition, individual practices are often shaped by structural and social variables, which can hinder behavioral change [29]. These include contextual challenges such as sleeping outdoors due to power outages, or limited access mosquito control materials such as mosquito coils, sprays, repellent cream, or ointments.

Our findings indicate that inclusion of dengue‐related subjects in university curricula positively influenced students' knowledge and attitudes. This observation aligns with previous studies carried out among students and youth in Dhaka city as well as findings from Riohacha in Colombia [20, 50, 51]. A plausible explanation is that education fosters behavioral changes resulting in changes in attitudes. Furthermore, university students possess higher levels of health literacy, equipping them to respond against dengue‐related risks [51]. Academic institutions therefore have a crucial role in health promotion, particularly in raising awareness among this vulnerable demographic. To sustain long‐term dengue prevention behaviors, education, and monitoring should continue [52].

In our study, female participants demonstrated lower attitude scores compared to their male counterparts a finding that contrasts with previous research conducted in Malaysia and Puerto Rico [53, 54]. This discrepancy may be attributable to the relatively smaller proportion of female participants in our study, which could have influenced a lower attitude level in this subgroup.

Our study identified a significant association between preventive practices and having family members affected by dengue. This result aligns with previous research conducted in Costa Rica, Pakistan, and Venezuela [34, 49, 55]. On the other hand, weaker association of preventive practices are observed among participants who reported dengue cases among their neighbors, a pattern similarly noted in a study conducted in Malaysia [56]. The possible discrepancy might be that respondents with household dengue infection may perceive dengue as heightened personal risk, more immediate and threatening. This perceived proximity likely serves as a strong motivator for adopting protective behaviors [34, 49, 55]. In contrast, lack of cues or support, inadequate community‐level awareness, preventive practices remain limited despite knowing dengue cases in neighboring households, engagement in protective practices often remains limited [56].

Our study identified a significant association between knowledge and the adoption of effective dengue‐related preventive practices among participants. These finding is in line with prior research demonstrating a positive correlation between dengue knowledge and the implementation of dengue prevention and control measures [34, 45, 53]. A plausible explanation is that knowledge helps individuals to recognize environmental barriers and devise strategies to mitigate them, thereby facilitating the adoption of feasible protective behaviors. This knowledge‐driven approach not only strengthens individual actions but also fosters collective efforts, amplifying the impact of prevention initiatives. Triandis emphasized that even when individual possess strong behavioral intention the successful enactment of those behaviors depends on adequate knowledge and skills [57, 58].

Our study demonstrated partial significant association between knowledge and attitude towards dengue fever. Despite partial significant association, the finding suggests that improving one or more components of KAP could help enhance the others among this learner group. Our finding aligns with previous studies conducted among university students residing in Dhaka city, studies conducted among students in South Asian countries [20, 59, 60].

However, our study found no significant association between attitudes and preventive practices toward dengue fever. A plausible explanation for this weak relationship may be the limited exposure of students to dengue cases during their academic tenure. Furthermore, many students reside away from their family homes and are less engaged in household level dengue prevention activities, which may hinder the development of strong preventive attitudes. Attitudes play a pivotal role when individuals perceive that influential figures such as family members, peers, or community leaders expect them to engage in preventive behaviors, and they are motivated to meet these expectations, they are more likely to develop favorable attitudes. This contributes to the formation of supportive subjective norms that reinforce preventive practices [58].

The study highlights that university students' engagement in preventive behaviors is influenced by their overall knowledge and attitudes toward dengue. Through nursing education, university authorities can enhance awareness and foster positive attitudes by providing guidance, resources, and programs that promote careful engagement in preventive behaviors. Systematic integration of infectious disease related subject and practical demonstrations is essential to adequately prepare this learner population. Through nursing clinical practice, the application of knowledge through practical measures such as promoting environmental hygiene, adopting protective measures, and raising awareness can help strengthen dengue prevention among university students.

### 4.1 Strengths and Limitations

To our knowledge, this is the first study to investigate KAP related to dengue fever university students of Bangladesh, the findings offer valuable insights into this understudied population and may serve as a baseline for future research on dengue trends in similar settings. However, the study has some drawbacks. The use of a convenience sampling technique and reliance on a self‐reported online survey may introduce reporting bias. Additionally, peer influence during survey completion could have affected the authenticity of responses. The exclusion of certain socio‐demographic variables further limits the generalizability of the findings.

## 5 Conclusion

Despite a generally positive attitude toward DF, our findings revealed inadequate levels of knowledge and preventive practices among university students. Universities can implement dengue focused awareness campaigns, during peak transmission seasons, incorporating educational sessions prevention and treatment. Sharing personal and community experiences may enhance student engagement and facilitate behavioral changes. Collaborative workshops with local health authorities and practical activities such as campus and neighborhood clean‐up drives can further reinforce preventive measures. Translating knowledge and attitudes into sustained preventive practices is essential for reducing risks among these vulnerable groups.

## Author Contributions

**N.G.U.:** conceptualization, methodology, validation, formal analysis, writing – original draft, writing – review and editing, data curation, and visualization. **P.C.:** methodology, formal analysis, and data curation. **T.H.:** writing – review and editing, and data curation. **S.C.D.:** writing – review and editing, and data curation. **S.M.:** writing – review and editing, and data curation. **M.B.A.:** formal analysis, writing – review and editing. **S.I.P.:** writing – review and editing, data curation. **M.H.:** writing – review and editing. **M.A.M.:** writing – review and editing. **M.H.R.:** validation, writing – original draft, final review, and editing. **M.N.H.:** validation, writing – original draft, final review, and editing.

## Funding

The authors received no specific funding for this work.

## Ethics Statement

Written consent was obtained from all participants prior to the interview. Research Ethical Committee (REC) of the Department of Environmental Sanitation, Patuakhali Science and Technology University, Bangladesh (approval no.: PSTU/IEC/2023/72) reviewed the protocol and approved this study.

## Conflicts of Interest

The authors declare no conflicts of interest.

## Transparency Statement

The corresponding author, Nasimul Ghani Usmani, affirms that this manuscript is an honest, accurate, and transparent account of the study being reported; that no important aspects of the study have been omitted; and that any discrepancies from the study as planned (and, if relevant, registered) have been explained.

## Supporting information

Supporting Material 1.docx.

Supporting Material 2.docx.

## Data Availability

The data sets generated during and/or analyzed during the current study are available from the corresponding author on reasonable request.
