# Supplementary material for: Exploring Knowledge, Attitudes, and Practices Regarding Dengue Fever Among University Students in Bangladesh: A Cross‐Sectional Study
Source: Health Sci Rep. 2025 Dec 30;9(1):e71714. doi: 10.1002/hsr2.71714 (PMC12754271; doi:10.1002/hsr2.71714)
Supplement: Supplementary file 1 — Supporting Material 1.docx. [file HSR2-9-e71714-s002.docx]

- **Appendix 01**

**Knowledge responses regarding dengue fever among participants (N= 671)**

| **Knowledge based questions** | **Correct response** | | **Incorrect**  **response** | |
| --- | --- | --- | --- | --- |
|  | **n** | **%** | **n** | **%** |
| 1. Is fever a symptom of dengue? | 607 | 90.5 | 63 | 9.39 |
| 2. Is headache a symptom of dengue fever? | 550 | 82 | 121 | 18 |
| 3. Is joint pain a symptom of dengue fever? | 587 | 87.5 | 84 | 12.5 |
| 4. Is muscle pain a symptom of dengue fever? | 527 | 78.5 | 144 | 21.5 |
| 5. Is pain behind the eyes a symptom of dengue fever? | 399 | 59.5 | 272 | 40.5 |
| 6. Are nausea/vomiting symptoms of dengue fever? | 413 | 61.6 | 258 | 38.5 |
| 7. Is rash a symptom of dengue fever? | 316 | 47.1 | 355 | 52.9 |
| 8. Is diarrhea common in dengue fever? | 219 | 32.6 | 452 | 67.4 |
| 9. Is back pain common in dengue fever? | 345 | 51.4 | 326 | 48.6 |
| 10. Is stomach pain common in dengue fever? | 191 | 28.5 | 480 | 71.5 |
| 11. Can all mosquitoes transmit dengue fever? | 619 | 92.3 | 52 | 7.8 |
| 12. Do Aedes mosquitoes transmit dengue fever? | 616 | 91.8 | 55 | 8.2 |
| 13. Do flies transmit Dengue fever? | 550 | 82 | 121 | 18 |
| 14. Do ticks transmit Dengue fever? | 448 | 66.7 | 223 | 33.2 |
| 15. Does ordinary person-to-person contact transmit Dengue fever? | 576 | 85.8 | 95 | 14.2 |
| 16. Is Dengue fever transmitted through food and water? | 514 | 76.6 | 157 | 23.4 |
| **17. Can Dengue fever be transmitted by blood transfusion?** | 323 | **48.1** | 348 | **51.9** |
| **18. When are Dengue mosquitoes likely to feed/bite?** | 236 | **35.2** | 435 | **64.8** |
| **(Options: 1. Night time, 2. Day time, 3. Both day and night, 4. Morning, 5. Evening, 6. Anytime, 7. Don’t know)** |  |  |  |  |
| 19. Mosquitoes breed in standing water. | 516 | 76.9 | 155 | 23.1 |
| 20. Window screens and bed nets reduce mosquitoes. | 518 | 77.2 | 153 | 22.8 |
| 21. Insecticide sprays reduce mosquitoes and prevent Dengue. | 518 | 77.2 | 153 | 22.8 |
| 22. Tightly covering water containers reduces mosquitoes. | 512 | 76.3 | 159 | 23.7 |
| 23. Removal of standing water can prevent mosquito breeding. | 562 | 83.8 | 109 | 16.2 |
| 24. Mosquito repellents prevent mosquitoes. | 486 | 72.4 | 185 | 27.6 |

**Attitude responses towards dengue fever among participants (N= 671)**

| **Attitude based questions** | Strongly agree  **n (%)** | Agree  **n (%)** | Neutral  **n (%)** | Disagree  **n (%)** | Strongly disagree  **n (%)** |
| --- | --- | --- | --- | --- | --- |
| Dengue fever is not a serious illness. | 206 (30.7%) | 179 (26.7%) | 71 (10.6%) | 110 (16.4%) | 105 (15.7%) |
| Are you at risk of getting dengue? | 128 (19.1%) | 218 (32.5%) | **220 (32.8%)** | **77 (11.5%)** | **28 (4.2%)** |
| Dengue fever can be prevented. | 224 (33.4%) | 333 (49.6%) | 79 (11.8%) | 21 (3.1%) | 14 (2.1%) |
| Controlling the breeding places of mosquitoes is a good strategy to prevent dengue fever. | 327 (48.7%) | 223 (33.2%) | 76 (11.3%) | 26 (3.9%) | 19 (2.8%) |
| Stagnant water around the houses in discarded tires, broken pots and bottles are breeding places of Aedes mosquitoes. | 362 (54.0%) | 189 (28.2%) | 63 (9.4%) | 34 (5.1%) | 23 (3.4%) |
| Communities should actively participate in controlling the vectors of Dengue. | 346 (51.6%) | 193 (28.8%) | 79 (11.8%) | 21 (3.1%) | 32 (4.8%) |

**Preventive practices responses towards dengue fever among participants (N= 671)**

| **Practice based questions** | **Good practice** | | **Poor practice** | |
| --- | --- | --- | --- | --- |
|  | **n** | **%** | **N** | **%** |
| Prevent mosquito-human contact? | 496 | 73.9 | 175 | 26.1 |
| Use insecticide sprays to reduce mosquitoes? | 425 | 63.3 | 246 | 36.7 |
| Use professional pest control to reduce mosquitoes? | 349 | 52.0 | 322 | 48.0 |
| Use screen windows to reduce mosquitoes? | 430 | 64.1 | 241 | 35.9 |
| Eliminate stagnant water around the house to reduce mosquitoes? | 566 | 84.4 | 105 | 15.7 |
| Cut down bushes in the yard to reduce mosquitoes? | 557 | 83.0 | 114 | 17.0 |
| Prevent water stagnation? | 548 | 81.7 | 123 | 18.3 |
| Use mosquito-eating fish to reduce mosquitoes? | 328 | 48.9 | 343 | 51.1 |
| Use mosquito coils to reduce mosquitoes? | 528 | 78.7 | 143 | 21.3 |
| Clean garbage/trash? | 580 | 86.4 | 91 | 13.6 |
| Dispose of water-holding containers such as tires, parts of automobiles, plastic bottles, cracked pots, etc.? | 564 | 84.1 | 107 | 16.0 |
| Use mosquito repellent/cream? | 409 | 61.0 | 262 | 39.1 |
| Use a fan? | 506 | 75.4 | 165 | 24.6 |
| Use smoke to drive away mosquitoes? | 408 | 60.8 | 263 | 39.2 |
| Covering the body with clothes? | 504 | 75.1 | 167 | 24.9 |
| Do nothing to reduce mosquitoes? | 471 | 29.9 | 200 | 29.89 |
| Eliminating mosquito breeding sites? | 531 | 79.1 | 140 | 20.9 |
| Cover water containers in the home? | 561 | 83.6 | 110 | 16.4 |
| Frequently clean water-filled containers and ditches around the house? Always 2. Often 3. Sometimes 4. Never 5. Don’t know | 446 | 66.5 | 225 | 33.5 |
| Does the government spray insecticides for controlling mosquitoes? | 480 | 71.54 | 191 | 28.5 |
| Do you turn containers upside down to avoid water collection? | 490 | 71.5 | 181 | 27.0 |

- **Appendix 02**

**Association between socio-demographic factors and dengue knowledge, attitudes and preventive practices (N= 671)**

| **Characteristics** | **knowledge** | | p value | **attitude** | | p  value | **practice** | | p value |
| --- | --- | --- | --- | --- | --- | --- | --- | --- | --- |
| **Gender** | High knowledge n (%) | Low knowledge n (%) |  | High  knowledge n (%) | Low  knowledge n (%) |  | High  knowledge n (%) | Low  knowledge n (%) |  |
| Male | 130 (28.6) | 324 (71.4) | 0.15 | 340 (74.9) | 114 (25.1) | **0.029** | 156 (34.4) | 298 (65.6) | 0.224 |
| Female | 74 (34.1) | 143 (65.9) |  | 145 (66.8) | 72 (33.2) |  | 85 (39.2) | 132 (60.8) |  |
| **Living with family** | | | | | | | | | |
| Yes | 87 (32.0) | 185 (68.0) | 0.462 | 193 (71.0) | 79 (29.0) | 0.527 | 105 (38.6) | 167 (61.4) | 0.231 |
| No | 117 (29.3) | 282 (70.7) |  | 292 (73.2) | 107 (26.8) |  | 136 (34.1) | 263 (65.9) |  |
| **Department/ Faculty/ Major** | | | | | | | | | |
| Arts and Social Sciences | 45 (30.4) | 103 (69.6) | 0.9 | 102 (68.9) | 46 (31.1) | 0.269 | 52 (35.1) | 96 (64.9) | 0.524 |
| Business Studies | 8 (26.7) | 22 (73.3) |  | 19 (63.3) | 11 (36.7) |  | 8 (26.7) | 22 (73.3) |  |
| Science and Engineering | 151 (30.6) | 342 (69.4) |  | 364 (73.8) | 129 (26.2) |  | 181 (36.7) | 312 (63.3) |  |
| **Dengue-relevant subject in the current curriculum** | | | | | | | | | |
| Yes | 109 (37.2) | 184 (62.8) | **0.001** | 225 (76.8) | 68 (23.2) | **0.022** | 109 (37.2) | 184 (62.8) | 0.541 |
| No | 95 (25.1) | 283 (74.9) |  | 260 (68.8) | 118 (31.2) |  | 132 (34.9) | 246 (65.1) |  |
| **Heard about dengue** | | | | | | | | | |
| Yes | 186 (29.2) | 451 (70.8) | **0.003** | 460 (72.2) | 177 (27.8) | 0.867 | 231 (36.3) | 406 (63.7) | 0.417 |
| No | 18 (52.9) | 16 (47.1) |  | 25 (73.5) | 9 (26.5) |  | 10 (29.4) | 24 (70.6) |  |
| **Dengue infection history in last six months** | | | | | | | | | |
| Yes | 21 (29.6) | 50 (70.4) | 0.873 | 55 (77.5) | 16 (22.5) | 0.302 | 23 (32.4) | 48 (67.6) | 0.513 |
| No | 183 (30.5) | 417 (69.5) |  | 430 (71.7) | 170 (28.3) |  | 218 (36.3) | 382 (63.7) |  |
| **House members infected with dengue in last six months** | | | | | | | | | |
| Yes | 76 (30.6) | 177 (69.4) | 0.874 | 193 (76.3) | 60 (23.7) | 0.071 | 108 (42.7) | 145 (57.3) | **0.004** |
| No | 128 (30.0) | 290 (70.0) |  | 292 (69.9) | 126 (30.1) |  | 133 (31.8) | 285 (68.2) |  |
| **Neighbours infected with dengue in last six months** | | | | | | | | | |
| Yes | 76 (37.4) | 127 (62.6) | **0.009** | 157 (77.3) | 46 (22.7) | 0.054 | 54 (26.6) | 149 (73.4) | **0.001** |
| No | 128 (27.4) | 340 (72.7) |  | 328 (70.1) | 140 (29.9) |  | 187 (40.0) | 281 (60.0) |  |

- **Appendix 03**

**List of universities from where data were collected**

**Public universities**

Patuakhali Science and Technology University; Barishal University; Rajshahi University; Chittagong University; Chittagong Veterinary and Animal Sciences University; Jahangirnagar University; University of Dhaka; Sylhet Agricultural University; Jashore University of Science and Technology; Islamic University; Bangladesh Agricultural University; Bangabandhu Sheikh Mujibur Rahman Science and Technology University; Cumilla University; Pabna University of Science and Technology; Hajee Mohammad Danesh Science and Technology University; Noakhali Science and Technology University; Shahjalal University of Science & Technology; Begum Rokeya University, Rangpur; Jatiya Kabi Kazi Nazrul Islam University

**Private universities**

Army Institute of Business Administration, Sylhet; Northern University of Business and Technology, Khulna; University of Global Village, Barishal; International University of Business Agriculture and Technology (IUBAT); Bangladesh University of Health Sciences; American International University-Bangladesh (AIUB); East West University; Daffodil International University; North South University; Independent University, Bangladesh; European University

**Appendix 04**

| **Characteristics** | **Adjusted R²** | **p-value** |
| --- | --- | --- |
| Knowledge | 21.1% | 0.013 |
| Attitude | 20.2% | 0.011 |
| Practice | 22.4% | 0.026 |

**Appendix 05**

**Variance Influence Factor (VIF) values**

| **Constructs** | **VIF** |
| --- | --- |
| Gender | 1.07 |
| Living with family | 1.10 |
| Residential unit | 1.08 |
| Department/ Faculty/ Major | 1.03 |
| Dengue-relevant subject in the curriculum | 1.07 |
| Dengue infection history in last six months | 1.13 |
| House members infected with dengue in last six months | 1.13 |
| Neighbours infected with dengue in last six months | 1.18 |
