# Supplementary material for: Exploring Knowledge, Attitudes, and Practices Regarding Dengue Fever Among University Students in Bangladesh: A Cross‐Sectional Study
Source: Health Sci Rep. 2025 Dec 30;9(1):e71714. doi: 10.1002/hsr2.71714 (PMC12754271; doi:10.1002/hsr2.71714)
Supplement: Supplementary file 2 — Supporting Material 2.docx. [file HSR2-9-e71714-s001.docx]

**Knowledge, Attitudes, and Practices (KAP) Questionnaire on Dengue Fever**

- **Appendix 01**

**Sociodemographic information**

| **Gender** | Male |
| --- | --- |
|  | Female |
| **Living with family** | Yes |
|  | No |
| **University type** | Public |
|  | Private |
| **Department/Faculty/Major** | Arts and Social Sciences |
|  | Business Studies |
|  | Science and Engineering |
| **Dengue-relevant subject in the curriculum** | Yes |
|  | No |
| **Heard about dengue** | Yes |
|  | No |
| **Dengue infection history in last six months** | Yes |
|  | No |
| **House members infected with dengue in last six months** | Yes |
|  | No |
| **Neighbours infected with dengue in last six months** | Yes |
|  | No |

**Knowledge based questions**

| 1 | Is fever a symptom of dengue? |
| --- | --- |
| 2 | Is headache a symptom of dengue fever? |
| 3 | Is joint pain a symptom of dengue fever? |
| 4 | Is muscle pain a symptom of dengue fever? |
| 5 | Is pain behind the eyes a symptom of dengue fever? |
| 6 | Are nausea/vomiting symptoms of dengue fever? |
| 7 | Is rash a symptom of dengue fever? |
| 8 | Is diarrhea common in dengue fever? |
| 9 | Is back pain common in dengue fever? |
| 10 | Is stomach pain common in dengue fever? |
| 11 | Can all mosquitoes transmit dengue fever? |
| 12 | Do Aedes mosquitoes transmit dengue fever? |
| 13 | Do flies transmit Dengue fever? |
| 14 | Do ticks transmit Dengue fever? |
| 15 | Does ordinary person-to-person contact transmit Dengue fever? |
| 16 | Is Dengue fever transmitted through food and water? |
| 17 | Can Dengue fever be transmitted by blood transfusion? |
| 18 | When are Dengue mosquitoes likely to feed/bite?  Options: 1. Night time, 2. Day time, 3. Both day and night, 4. Morning, 5. Evening, 6. Anytime, 7. Don’t know) |
| 19 | Mosquitoes breed in standing water. |
| 20 | Window screens and bed nets reduce mosquitoes. |
| 21 | Insecticide sprays reduce mosquitoes and prevent Dengue. |
| 22 | Tightly covering water containers reduces mosquitoes. |
| 23 | Removal of standing water can prevent mosquito breeding. |
| 24 | Mosquito repellents prevent mosquitoes. |

**Attitude based questions**

|  |  | Strongly agree | Agree | Neutral | Disagree | Strongly disagree |
| --- | --- | --- | --- | --- | --- | --- |
| 1 | Dengue fever is not a serious illness. |  |  |  |  |  |
| 2 | Are you at risk of getting dengue? |  |  |  |  |  |
| 3 | Dengue fever can be prevented. |  |  |  |  |  |
| 4 | Controlling the breeding places of mosquitoes is a good strategy to prevent dengue fever. |  |  |  |  |  |
| 5 | Stagnant water around the houses in discarded tires, broken pots and bottles are breeding places of Aedes mosquitoes. |  |  |  |  |  |
| 6 | Communities should actively participate in controlling the vectors of Dengue. |  |  |  |  |  |

**Preventive practices-based questions**

| 1 | Prevent mosquito-human contact? |
| --- | --- |
| 2 | Use insecticide sprays to reduce mosquitoes? |
| 3 | Use professional pest control to reduce mosquitoes? |
| 4 | Use screen windows to reduce mosquitoes? |
| 5 | Eliminate stagnant water around the house to reduce mosquitoes? |
| 6 | Cut down bushes in the yard to reduce mosquitoes? |
| 7 | Prevent water stagnation? |
| 8 | Use mosquito-eating fish to reduce mosquitoes? |
| 9 | Use mosquito coils to reduce mosquitoes? |
| 10 | Clean garbage/trash? |
| 11 | Dispose of water-holding containers such as tires, parts of automobiles, plastic bottles, cracked pots, etc.? |
| 12 | Use mosquito repellent/cream? |
| 13 | Use a fan? |
| 14 | Use smoke to drive away mosquitoes? |
| 15 | Covering the body with clothes? |
| 16 | Do nothing to reduce mosquitoes? |
| 17 | Eliminating mosquito breeding sites? |
| 18 | Cover water containers in the home? |
| 19 | Frequently clean water-filled containers and ditches around the house? Always 2. Often 3. Sometimes 4. Never 5. Don’t know |
| 20 | Does the government spray insecticides for controlling mosquitoes? |
| 21 | Do you turn containers upside down to avoid water collection? |
